# “Mind the Gap”—enlarged perivascular spaces as a potential magnetic resonance imaging biomarker of impaired glymphatic clearance in brain disorders

**DOI:** 10.3389/fncel.2026.1741115

**Published:** 2026-04-01

**Authors:** Claudia F. Kirsch, Mackenzie Herb, Guarav Verma, Priti Balchandani

**Affiliations:** 1 Department of Radiology and Biomedical Imaging, School of Medicine, Yale University, New Haven, CT, United States; 2 Yale School of Medicine, New Haven, CT, United States; 3 Icahn School of Medicine at Mount Sinai, New York, NY, United States

**Keywords:** artificial intelligence, autism spectrum disorder, enlarged perivascular spaces (ePVS), glymphatic system, magnetic resonance imaging (MRI), neurological disease, perivascular space (PVS)

## Abstract

The abundant capillary network penetrating the brain parenchyma is surrounded by potential tubular, fluid-filled regions referred to as perivascular spaces (PVSs). PVSs have a unique and complex history and are believed to act as a pathway for the drainage of waste products from brain interstitial and cerebrospinal fluid (CSF) as part of the glymphatic clearance system. The unique perivascular “gap” spaces are eponymously linked to Virchow and Robin, who argued vigorously in the 1800s over PVSs’ exact location and physiology. Currently, debates are ongoing regarding whether PVSs are predominantly periarteriolar, perivenular, or both and how they aid in clearing fluids from the brain parenchyma. In neurodevelopmental, neuropsychiatric, and neuropathological conditions, PVS can enlarge, a phenomenon referred to as enlarged perivascular spaces (ePVSs), which are identifiable on magnetic resonance imaging (MRI), with improved detection and resolution at higher magnetic field strengths. Quantification of ePVS enlargement on MRI using artificial intelligence (AI) imaging algorithms may serve as a potential non-invasive imaging biomarker for impaired glymphatic clearance and brain disorders. This mini-review presents the historical background and pathophysiology of PVSs and ePVSs, current debates regarding their exact location, their potential as neuroimaging biomarkers, and how AI may aid in ePVS quantification.

## Introduction

The brain is highly perfused by a vast arterial, venous, and capillary network measuring up to approximately 650 kilometers (Zlokovic and Apuzzo, 1998; Duvernoy, 1999). The abundant capillary network penetrates the brain parenchyma and is surrounded by potential tubular, fluid-filled spaces referred to as perivascular spaces (PVSs). PVSs have a unique and complex history and are believed to be a drainage pathway for brain waste products in cerebrospinal fluid (CSF) and interstitial fluid (IF) via the glymphatic clearance system (Yu et al., 2022). In addition, PVSs contain perivascular immune cells, distinct from pericytes and microglia, which may be affected in inflammatory conditions (Kida et al., 1993).

PVSs are generally thought to represent fluid-filled gap spaces between endothelial cells and astrocytes that follow the penetrating brain vessels. The glymphatic system is theorized to facilitate the removal of metabolic waste from the brain via IF and CSF, functioning predominantly during sleep (Plog and Nedergaard, 2018). Currently, PVSs are believed to be involved in clearing brain molecular debris via IF and CSF as part of solute waste clearance (Iliff et al., 2012). When PVSs enlarge and are identifiable on brain magnetic resonance imaging (MRI), they are referred to as enlarged PVSs (ePVSs) (Marín-Padilla and Knopman, 2011; Nelson et al., 1961), suggesting impaired PVS function and observed across multiple neurological disorders (Al Abdulsalam et al., 2018; Barisano et al., 2021b; Cheng et al., 2025; Cho et al., 2025; Maclullich et al., 2004; Ma et al., 2025; Rudie et al., 2018; Salzman et al., 2005; Vinters et al., 2018). Quantification of ePVS on MRI may serve as a radiographic biomarker of impaired glymphatic waste clearance (Benveniste et al., 2019; Gouveia-Freitas and Bastos-Leite, 2021; Hayden and Tyagi, 2025; Huang et al., 2021; Iliff et al., 2012; Jie et al., 2020; Pollock et al., 1997; Solé-Guardia et al., 2025; Yu et al., 2022). This mini-review presents the historical background and controversies regarding PVS anatomical locations, current PVS anatomical and physiological models, the potential of ePVS to serve as a neuroimaging biomarker of impaired clearance in brain disorders, and the role of artificial intelligence (AI) in ePVS quantification (Oltmer et al., 2024; Ringstad, 2024).

## Historical background

Although PVSs are eponymously referred to as Virchow–Robin spaces (VR), these unique spaces or “gaps” were first described by Durand-Fardel in 1842 in his “Memoir on Softening of the Brain” (Durand-Fardel, 1843). Fardel noted that post-mortem brains had numerous small holes with a “sieve-like” appearance in the lentiform nuclei and white matter, which he termed “etat crible” or “status cribrosum.” The expression “passer au crible,” meaning to put something through a fine-tooth comb, arguably mirrors the goal of this mini-review: to scrutinize how ePVS are identified on MRI (Durand-Fardel, 1843).

Debates and controversies regarding the exact location and physiological function of PVSs date back to Virchow and Robin (Pestalozzi, 1849). In 1851, Virchow analyzed pathologic specimens and confirmed a sub-adventitial space between a vessel’s outer lamina tunica adventitia, inner tunica intima, and middle lamina media, continuous with capillaries. He termed this a dissecting ectasia (“disseziierende Ektasie”) and proposed that this space communicated directly with the subarachnoid space, allowing for brain fluid and waste clearance (Virchow, 1851). Virchow’s student, Wilhelm His, believed PVSs were analogous to the body’s lymphatic system (His, 1865).

In 1859, Charles Philippe Robin confirmed Virchow’s findings and was the first to describe PVSs as normal channels (Robin, 1859). However, Robin strongly disagreed with Virchow, arguing that PVSs were intra-adventitial, closed spaces connected to peri-neuronal areas (Jochems et al., 2020; Liu W. et al., 2025; Ma et al., 2025; Potter et al., 2015). Although Virchow and Robin vigorously argued about the anatomy and function of PVSs, their names are eponymously linked to PVSs for eternity.

In the 1900s, research on rodent brains examined how Prussian blue dye tracked from the subarachnoid space into the PVS (Weed, 1914; Schirge et al., 2025; Smeijer et al., 2019; Zabel et al., 2013; Zhu et al., 2010, 2011; Perosa et al., 2022). It was not until the 1970s that PVSs were visualized non-invasively in the brain, due to the development of MRI for clinical use (Damadian et al., 1974; Dreizen, 2004; Damadian et al., 1977; Lauterbur, 1989). In the 1980s, PVSs and ePVSs were described as linear fluid spaces extending parallel to the perforating vessels in the midbrain, hippocampus, lentiform nuclei, and white matter in the centrum semiovale (Braffman et al., 1988).

## Anatomical location and physiology

**Figure 1:**
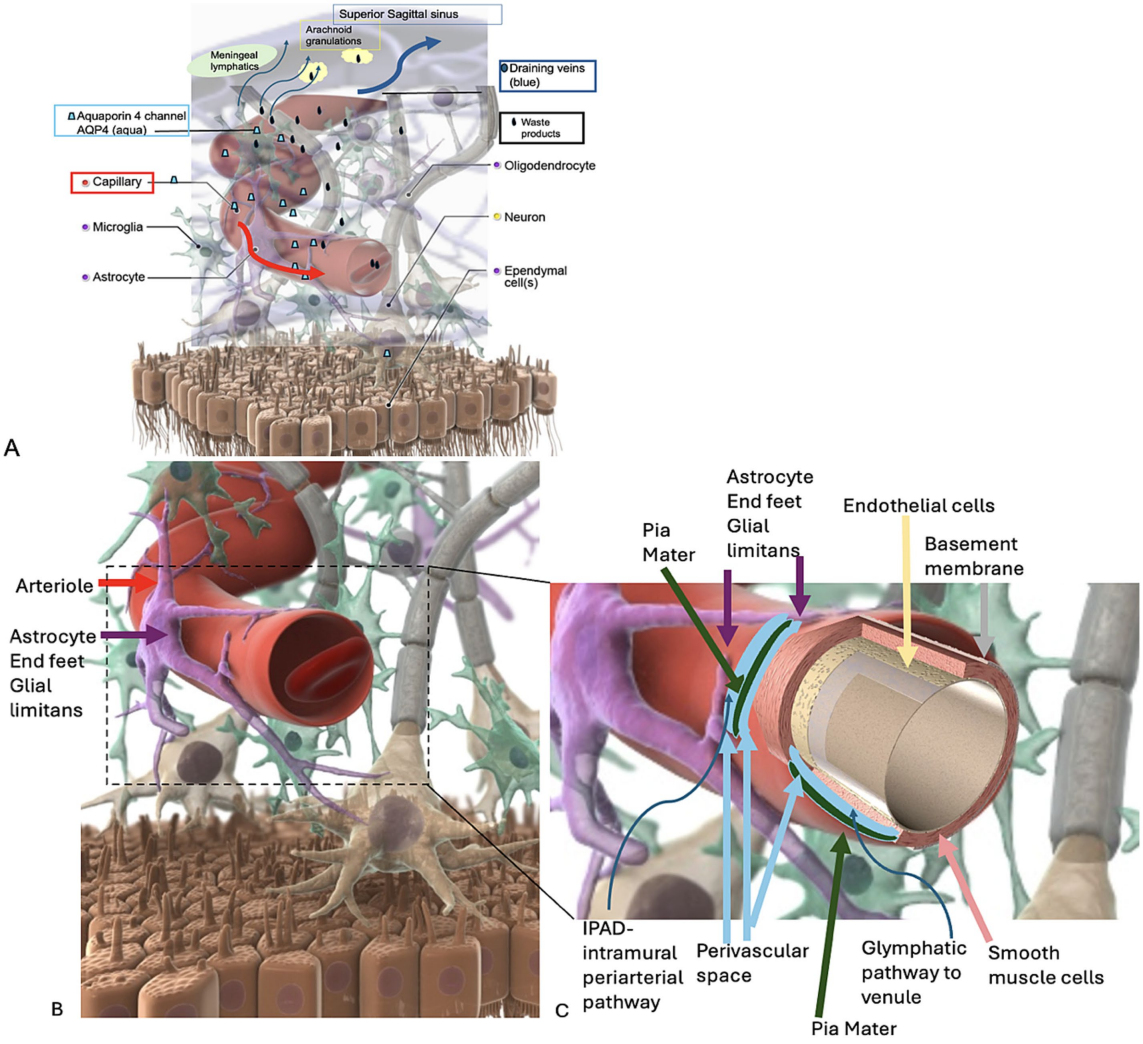
Schematic representation of brain clearance pathways. **(A)** The proposed glymphatic pathway (red arrow) drains cerebrospinal fluid (CSF) and solutes into the brain parenchyma. Aquaporin-4 channels (AQP4) on astrocytic endfeet facilitate fluid entry. Interstitial fluid (IF) and waste solutes exit via perivenous pathways (blue arrow). **(B,C)** Enlarged view of the intramural periarterial drainage (IPAD) pathway. Here, waste material exits via the basement membrane, which is interwoven with smooth muscle cells along arterioles and arteries, distinct from glymphatic flow. Images edited and utilized with permission from Primal Pictures—Anatomy.TV C. Kirsch MD.

Anatomically, PVSs are believed to be fluid-filled regions between endothelial cells and astrocytes that extend along brain arterioles, arteries, veins, and venules. They are separated from the brain by a thin glial cell layer and are lined by the pia mater (Albargothy et al., 2018). At the arteriole level, PVSs are contained by the pia mater; as the vessel decreases in size to a capillary, the pia mater is replaced by astrocytic endfeet (Figure 1). There are two primary models proposed for how PVSs may be involved in brain waste clearance: the “glial cell mediated lymphatic” (glymphatic) model and the “intramural periarterial drainage” (IPAD) model. In the glymphatic pathway model, PVSs are believed to be located anatomically along arterioles between the pia mater and smooth muscle cells and along venules between the pia mater (acting as an outer PVS wall) and the endothelium (Figure 1A) (Jessen et al., 2015). Iliff et al. (2012) utilized *in vivo* two-photon imaging of fluorescent tracers to propose that CSF enters the brain via cortical pial arteries, followed by influx into the PVS surrounding penetrating arterioles. This model posits that CSF is driven by arterial pulsation and vasomotion to enter the brain parenchyma via aquaporin-4 (AQP4) water channels on perivascular astrocytic endfeet. The CSF then mixes with IF and brain metabolic waste, diffuses through the parenchyma, and exits via para-venous spaces.

In the IPAD model (Figure 1B), CSF flows along the PVS of arteries and arterioles, located between the pia mater and glia limitans. However, in this pathway, waste material exits via the basement membrane, which is interwoven with smooth muscle cells along arterioles and arteries (Carare et al., 2008). Despite these proposed mechanisms, debates persist regarding the predominant location of PVS (periarteriolar vs. perivenular) (Smets et al., 2024; Brown et al., 2002; Taoka et al., 2017, 2024; Zhang et al., 2025). Although animal studies suggest that AQP4 channels are crucial for waste clearance, showing an approximately 70% reduction in interstitial solute clearance in their absence, MRI currently lacks the resolution to fully distinguish between periarteriolar and perivenular spaces (Abbrescia et al., 2024; Mestre et al., 2017; Liu et al., 2022).

## Clinical associations: neurological and psychiatric disorders

Findings of ePVSs have been reported in multiple neurological disorders, including stroke and small vessel disease (Solé-Guardia et al., 2025; Bown et al., 2022; Brown et al., 2018; Cavallari et al., 2018; Coleman et al., 2024; Doubal et al., 2010; Jochems et al., 2020; Langan et al., 2022), systemic lupus erythematosus (SLE) (Miyata et al., 2017), mild traumatic brain injury (Inglese et al., 2005), Parkinson’s disease (Firouzabadi et al., 2025; Zarkali, 2025), and Alzheimer’s dementia (Menze et al., 2024; Banerjee et al., 2017; Hong et al., 2024; Koo et al., 2016; Perosa et al., 2022). More recently, ePVSs have been identified in neurodevelopmental and neuropsychiatric disorders, with increased ePVS burden reported in children with autism spectrum disorder (ASD), correlating with the severity of neurodevelopmental symptoms (Frigerio et al., 2025; Sotgiu et al., 2025). In infancy, ePVSs have been associated with later sleep problems and autism diagnosis, potentially linking sleep-dependent glymphatic clearance mechanisms to neurodevelopment (Garic et al., 2023). Furthermore, ePVSs have been observed in neuropsychiatric conditions. For example, Mudalige et al. (2025) found associations between ePVSs and mild behavioral impairment (MBI) in older adults. Interestingly, Li et al. (2025) reported ePVSs in young adults with anxiety and depression associated with prolonged mobile phone use. These findings suggest that impaired glymphatic clearance may be a shared pathophysiological feature across a spectrum of brain disorders.

## MRI visualization and measurement of PVSs and ePVSs

**Figure 2:**
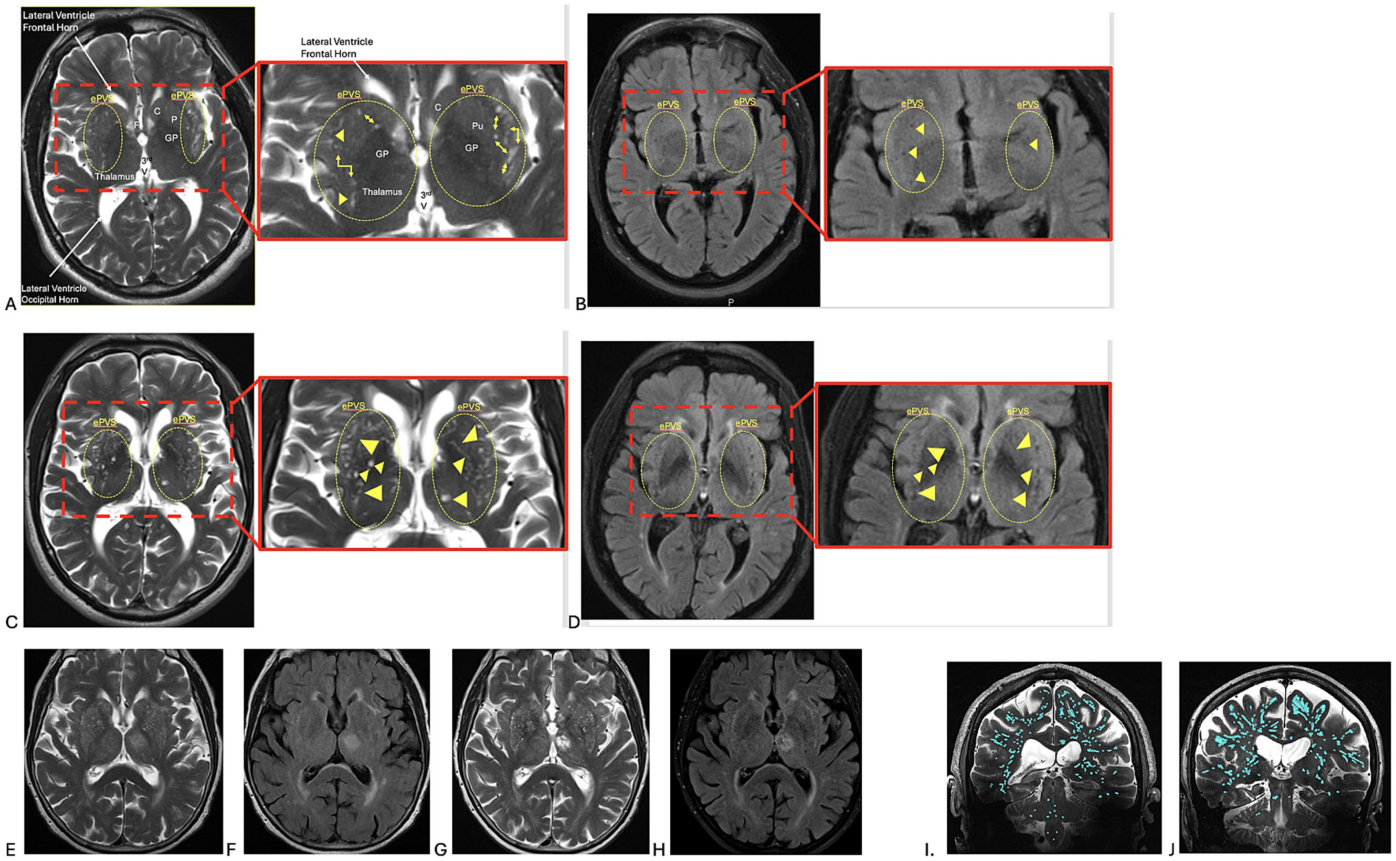
**(A,B)** Longitudinal MRI of a 65-year-old male individual with Alzheimer’s dementia on 1.5 T MRI. **(A)** Axial T2 and **(B)** axial FLAIR images at baseline. A total of 10 years later, the same patient is shown on **(C)** axial T2 and **(D)** axial FLAIR images, now demonstrating enlarged perivascular spaces (ePVSs) (highlighted within yellow dotted lines and indicated by yellow arrows and arrowheads) in the basal ganglia and periventricular white matter. The ePVSs are markedly enlarged on scans obtained 10 years later **(C,D)**. They appear hyperintense on T2-weighted sequences with fluid content and hypointense on FLAIR sequences. In contrast, white matter microvascular disease, unlike PVSs, is hyperintense on both T2- and FLAIR-weighted sequences. AC, anterior commissure; ACA, anterior cerebral artery; C, caudate; ePVS, Enlarged perivascular space; F, fornix; GP, globus pallidus; MCA, middle cerebral artery; MTT, mammillothalamic tract; PC, posterior commissure; PCFF, posterior commissural fibers of the fornix; PVS, perivascular space. **(E–H)** An 85-year-old female individual with vascular dementia on 3 T brain MRI. **(E,F)** Axial T2-weighted and FLAIR images demonstrate multiple foci of white matter hyperintensity, likely sequelae of microvascular disease, along with ePVSs in the lentiform nuclei, thalami, and periventricular white matter, exhibiting an “etat crible” appearance. A total of 2 months later, after contracting COVID-19, the same patient is shown in **(G,H)**, demonstrating persistent white matter microvascular disease along with increased size of multiple ePVSs in the lentiform nuclei, thalami, and periventricular white matter. **(I,J)** Ultra-high field 7 T MRI coronal T2-weighted images, with enlarged perivascular spaces (ePVSs) highlighted in light blue: **(I)** a healthy control and **(J)** a similarly aged patient with mild cognitive impairment demonstrating prominent ePVSs.

The best current method for visualizing PVSs and ePVSs *in vivo* is MRI. On 1.5 T or 3 T MRI, ePVSs are classically seen in the lentiform nuclei along the lenticulostriate arteries, as well as in the internal and external capsules and the white matter of the centrum semiovale. On MRI, ePVSs appear as T2 hyperintense fluid signals, predominantly along arterioles. When viewed in the same plane running parallel to brain vessels, they appear as linear hyperintense foci; in perpendicular views (“en face”), they appear as rounded hyperintense T2 foci. The “etat crible” appearance of ePVSs is observed more frequently in patients with Alzheimer’s dementia compared to healthy controls (Hansen et al., 2015; Kwee and Kwee, 2007; Patankar et al., 2005). Figures 2A–D demonstrates the progression of ePVSs in a patient with Alzheimer’s dementia over 10 years on a 1.5 T MRI. Figures 2E–H illustrates the progression of ePVSs in a patient with vascular dementia following COVID-19 infection on a 3 T MRI. Accurate measurement of PVSs and ePVSs is influenced by MRI field strength. Although ePVSs are visible on 1.5 T and 3 T MRI, as shown in Figures 2A–H, Valdés Hernández et al. (2025) demonstrated that a greater number of ePVSs were detected on 3 T MRI compared to 1.5 T MRI. However, harmonization techniques could compensate for this measurement bias. As MRI field strength increases, especially with ultra-high field (UHF) 7 T scanners, the accuracy of quantitative volume and caliber measurements of ePVSs improves (Barisano et al., 2021a; Bouvy et al., 2014; Feldman et al., 2018; Liu S. et al., 2025; Ranti et al., 2022; Sepehrband et al., 2017; Spijkerman et al., 2022; Zong et al., 2016). This is demonstrated in the coronal T2 images comparing similarly aged patients, with an increased number of ePVSs, highlighted in light blue, noted in the patient with mild cognitive impairment (Figures 2I,J).

Multiple grading scales exist for evaluating ePVSs, such as the Wardlaw scale, which rates PVS burden in the basal ganglia and centrum semiovale on a 5-point scale (Potter et al., 2015; González-Castro et al., 2017; Paradise et al., 2020). As manual counting is tedious and may miss asymmetric distributions, as seen in stroke or epilepsy, ePVS assessment is now performed through techniques utilizing artificial intelligence (AI) and machine learning (ML) for quantification (Pham et al., 2022; Rashid et al., 2023). Methods for AI-based ePVS assessment include a semi-automated segmentation approach developed by Smith et al. (2020), termed PVSSAS, which applies Frangi filters to 7 T MRI data. A meta-analysis by Waymont et al. (2024) confirmed that morphological filters (such as Frangi) and U-Net configurations are the most widely used automated methods.

## Conclusion

This mini-review began with the historical discovery of the unique potential gap spaces known as perivascular spaces (PVSs). Their exact location and function sparked debates between Virchow and Robin, whose names remain eponymously linked to PVSs. Enlargement of PVSs has been associated with multiple brain pathologies, ranging from neurodegenerative diseases such as Alzheimer’s disease to neurodevelopmental conditions such as ASD. As MRI techniques and AI continue to improve the detection and quantification of ePVSs, these tools may eventually resolve unanswered questions regarding their exact location and their role as biomarkers of impaired glymphatic clearance.
